# Identifying a Population Living With Alzheimer’s: Concordance Between Medicare Claims and Survey Reports

**DOI:** 10.1093/geroni/igaa057.559

**Published:** 2020-12-16

**Authors:** Ayse Akincigil, Camille McKenzie

**Affiliations:** 1 Rutgers, The State University of New Jersey, New Brunswick, New Jersey, United States; 2 Rutgers University--New Brunswick, New Brunswick, New Jersey, United States

## Abstract

Community-level estimates of Alzheimer’s disease and related dementias (ADRD) are necessary to assess health care needs and supports (to patients and family members), determine the burden of disease, conduct public health planning, improve access and care quality improvement, and to build a workforce with the necessary skills. Data from Medicare claims can provide efficient and timely estimates. However, earlier studies suggest that identifying ADRD populations solely from Medicare claims fails to capture many individuals that live with ADRD, with false-negative cases as high as 60%. We examined nationally representative data from the 2015-2017 Medicare Current Beneficiary Survey (MCBS) to assess the claims-based case ascertainment method, covering the transition to the International Classification of Diseases, Tenth Revision (ICD-10). The study population included community dwellers aged 65 or older, enrolled in traditional fee-for-service (n=12,409). Claims based method identified 1,325 cases (10.7% prevalence). However, there were 196 (1.6%) additional cases that self/proxy reported ADRD, but there was no ADRD diagnosis in any of their Medicare claims (hereafter referred to as self-report only). On average, the self-report only group reported higher numbers of limitations in activities, or instrumental activities of daily living, worse overall health, and more difficulty in concentrating or remembering, suggesting they are likely to be false negatives under claims-based case ascertainment method. In conclusion, claims based case ascertainment methods failed to capture some individuals with ADRD, but the magnitude of false-negative cases declined substantially in the era of ICD-10.

